# Demystifying the Role of Prognostic Biomarkers in Breast Cancer through Integrated Transcriptome and Pathway Enrichment Analyses

**DOI:** 10.3390/diagnostics13061142

**Published:** 2023-03-16

**Authors:** Divya Mishra, Ashish Mishra, Sachchida Nand Rai, Emanuel Vamanu, Mohan P. Singh

**Affiliations:** 1 Centre of Bioinformatics, Institute of Interdisciplinary Studies, University of Allahabad, Prayagraj 211002, India; 2 Faculty of Biotechnology, University of Agricultural Sciences and Veterinary Medicine, 011464 Bucharest, Romania; 3 Centre of Biotechnology, Institute of Interdisciplinary Studies, University of Allahabad, Prayagraj 211002, India

**Keywords:** gene, molecular diagnostic, prognosis, therapy, database

## Abstract

Breast cancer (BC) is the most commonly diagnosed cancer and the leading cause of death in women. Researchers have discovered an increasing number of molecular targets for BC prognosis and therapy. However, it is still urgent to identify new biomarkers. Therefore, we evaluated biomarkers that may contribute to the diagnosis and treatment of BC. We searched TCGA datasets and identified differentially expressed genes (DEGs) by comparing tumor (100 samples) and non-tumor (100 samples) tissues using the Deseq2 package. Pathway and functional enrichment analysis of the DEGs was performed using the *DAVID (Database* for Annotation, Visualization, and Integrated Discovery*) database*. The protein–protein interaction (PPI) network was identified using the STRING database and visualized through Cytoscape software. Hub gene analysis of the PPI network was completed using cytohubba plugins. The associations between the identified genes and overall survival (OS) were analyzed using a Kaplan–Meier plot. Finally, we have identified hub genes at the transcriptome level. A total of 824 DEGs were identified, which were mostly enriched in cell proliferation, signal transduction, and cell division. The PPI network comprised 822 nodes and 12,145 edges. Elevated expression of the five hub genes *AURKA, BUB1B, CCNA2, CCNB2,* and *PBK* are related to poor OS in breast cancer patients. A promoter methylation study showed these genes to be hypomethylated. Validation through genetic alteration and missense mutations resulted in chromosomal instability, leading to improper chromosome segregation causing aneuploidy. The enriched functions and pathways included the cell cycle, oocyte meiosis, and the p53 signaling pathway. The identified five hub genes in breast cancer have the potential to become useful targets for the diagnosis and treatment of breast cancer.

## 1. Introduction

Breast cancer (BC) is the most common type of cancer and the second most prominent cause of cancer-related death in women [1]. According to the World Health Organization (WHO), in 2020, there were 2.3 million women diagnosed with breast cancer and 685,000 deaths globally [2]. The lack of improved adjuvant therapy is also a major problem in reducing the burden of BC patients. Currently, the lymph node involvement, tumor size, and distant metastasis of the American Joint Committee on Cancer have been extensively identified, but there is still a need for a globally recognized platform or efficient markers that can correctly predict the prognosis of BC patients [3]. Even though applying for endocrine therapy or neoadjuvant chemotherapy, clinic-pathological parameters are commonly ambiguous, which complicates the judgments of real prognosis [4]. Approximately 70–80% of BC patients can be cured, especially when the disease is identified early, while advanced BC having distant organ metastases is considered incurable with currently available treatment strategies. Therefore, there is a critical need to find breast cancer biomarkers that can help to develop better treatment strategies for breast cancer. Comprehensive research is required to focus on understanding the molecular basis of BC [5].

Since then, many genes have been identified as prognostic and predictive biomarkers of breast cancer that play a significant role in precise treatment [6,7]. The commonly targeted drugs used for HER2-positive BC include trastuzumab, lapatinib, tucatinib, trastuzumab emtansine (T-DM1) and pertuzumab. Many molecular-targeted drugs therapy include the mammalian target of rapamycin (mTOR)/serine/threonine kinase (AKT)/phosphoinositide 3-kinase (PI3K) signaling pathways, which include bupacoxib, abencoxib, GDC-0068, alpelisib, and Bez235 [1]. Therefore, vascular endothelial growth factor has found to be as a key target for anti-angiogenic treatment, and its reported inhibitors such as sorafenib, sunitinib, and bevacizumab are being utilized for breast cancer therapy [6]. Androgen receptor (AR)-based targeted therapies can include AR antagonists and AR agonists which showing prominent results in clinical trials for BC patients [8].

Likewise, the combinations of AR-based targeted treatments with other reagents such as PI3K inhibitor have been analyzed to overcome resistance to AR-targeted treatments. In contrast, the targeted treatment strategies have been extensively developed for cyclin-dependent kinase 4/6 (CDK4/6), *BRCA1/2*-mutated polyadenosine diphosphate ribose polymerase (PARP), BTB and CNC homology 1 (BACH1), epidermal growth factor receptor (EGFR), and so on. However, due to low ratios of responders, tumor heterogeneity, and drug resistance, there is still a strong need to identify new biomarkers that can help diagnose and treat BC [1].

Computational analysis is one of the efficient strategies for the comprehensive study of large databases that include complex genomic information [9]. Our present study used sophisticated in silico approaches to identify potential prognostic biomarkers that can be useful for BC. Therefore, this analysis includes the identification of differentially expressed genes that were overexpressed in BC. The five hub genes obtained were further validated through promoter methylation, mutation and genetic alterations analysis, which proved their potential to be prognostic biomarkers. The survival analysis of all these hub genes showed poorer survival rates among BC patients.

## 2. Materials and Methods

### 2.1. Fetching and Preprocessing of Data and Determination of Differentially Expressed Genes through DESeq2 Analysis

The raw data for the solid normal samples and primary tumor were obtained from The Cancer Genome Atlas (TCGA). The raw data were pre-processed using bioinformatics tools and software. The quality assessment of the raw reads was carried out using FastQC (v 0.11.8) to identify the short length reads (adapter content) having low quality and uncalled biases. The low-quality reads were filtered and trimmed using Cutadapt software tool (v 3.2) for removing the noise in the data that could affect the results drastically. The trimmed reads were further aligned against the human reference genome (GRch38/hg38) using the STAR alignment tool (v 2.7.7a) and is considered as one of the fastest global alignment tools [10]. In the next step, the mapped reads were quantified to obtain the read counts corresponding to each gene through featureCounts (v 2.0.1) [11] Finally, the differentially expressed genes (DEGs) were obtained between solid normal samples and primary tumors through DESeq2 (v 1.22.1), which provided the quantitative variation in the expression levels of genes. This process is based on the normalization of the data using negative binomial distribution [12]. The criteria specified for categorizing the genes as significantly differentially expressed were the false discovery rate (*p*-value (adj.) < 0.05) and |log_2_FC| > 2.

**Figure 1:**
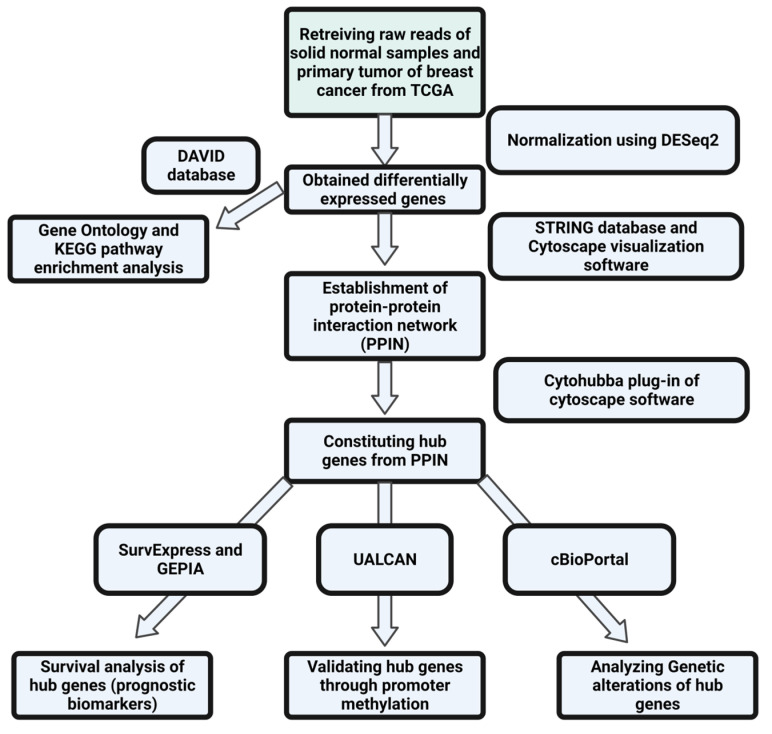
Flowchart showing the methodology followed in the present study.

The flowchart shown below depicts the entire process that was followed in this study (Figure 1).

### 2.2. Investigating the Protein–Protein Interaction Network (PPIN) to Establish the Hub Genes as Potential Prognostic Biomarkers

The protein–protein interaction network deals with mathematical representations pertaining to physical contacts established between different cellular level proteins and is crucial for understanding the processes that are taking place at the cellular level in normal and diseased states. The STRING database developed for the purpose of constructing the PPI network was used in this case, and this database uses the differentially expressed genes as input to provide the required result [13]. The nodes of the network correspond to differentially expressed genes (DEGs), and the edges constitute the interaction between the proteins. Cytoscape visualization software was used to visualize the various interactions and analyze the PPI network [14]. The significance of the interactions in the PPI network was analyzed through PPI enrichment value < 1.0 × 10^−16^. A confidence interval <0.4 was set for constructing the PPIN. For determining the hub genes as prognostic biomarkers, the cytohubba plug-in, available in the Cytoscape software, was used. Overall, 6 significant topologies of cytohubba viz. Degree, Maximal Clique Centrality (MCC), Maximum Neighborhood Component (MNC), Edge Percolated Component (EPC), Radiality, and Closeness were employed. From these five algorithms, the hub genes common among all of these were finally established using the jVenn online tool [15].

### 2.3. Analyzing the Gene Ontology (GO) Components and Enriched Pathways Involved in the Progression of Breast Cancer 

DAVID (Database for Annotation, Visualization and Integrated Discovery) is an online tool for establishing the functional enrichment of overexpressed genes involved in different disease types [16]. In the case of the present study, the gene list was uploaded in the database for exploring both GO terms and KEGG pathways involved in breast cancer. The modified Fisher exact *p*-value was set to 0.1, and this value aided in the measurement of gene enrichment in annotation terms. Likewise, the value for count threshold was fixed at 2, and this is the default value in the database. The lesser value of *p*-value indicates more enriched GO terms and KEGG pathways. These terms are considered significant based on the cut-off value for any term or pathway, which was set at *p* < 0.05. For visualizing these obtained components from DAVID, an online server, REVIGO [17], was used. It provided the treemaps corresponding to biological processes, cellular components and molecular functions based on the GO IDs and respective *p*-values of each component.

### 2.4. Exploring the Epigenetic Regulation of Hub Genes through Promoter Methylation 

The analysis of the consequences on the overexpressed genes due to the variations at the epigenetic level provides an in-depth knowledge about the tumorigenesis and metastasis of breast cancer. The promoter methylation study provides this information, and it can be obtained for each gene through an online server, UALCAN [18]. This multi-omics server dedicated to cancer study employs TCGA datasets, and for the analysis of the present study, datasets related to breast cancer were employed. The result could be interpreted based on the beta values that indicate the level of DNA methylation. These values range from 0 (unmethylated) to 1 (fully methylated). The beta values ranging between 0.5 and 0.7 pertain to hypermethylation, while those between 0.05 and 0.3 correspond to hypomethylation. 

### 2.5. Identifying the Genetic Alterations of Hub Genes

Different external and internal factors are responsible for causing genetic alterations such as mutations and copy number alterations, and these alterations result in altering the DNA sequences and play a pivotal role in the development and progression of cancer, its metastasis and providing resistance to therapies. In the present study, these genetic alterations in the hub genes were identified using the cBioPortal online resource, which contains genomic datasets of patients suffering from different cancer types [19]. The results pertaining to copy number alterations were obtained from GISTIC (Genomic Identification of Significant Targets in Cancer) algorithms, which identify the significantly altered regions across the different sets of patients. These results obtained from GISTIC correspond to the level of copy number per gene where a value of −2 indicates deep deletion or deep loss and constitutes homozygous deletion. Similarly, a value of −1 corresponds to shallow deletion and constitutes a heterozygous deletion. The value 0 corresponds to normal or diploid, 1 corresponds to gain (low-level gain) and 2 corresponds to amplification (high-level amplification). For visualizing these alterations (mutations and copy number alterations) obtained for different hub genes, OncoPrints was used. The mutations that occurred in the intronic region referred to splice site mutation, while those that occurred at the exon/intron junction referred to splice region mutations. 

### 2.6. Validating the Differential Expression Pattern and Survival Analysis of Hub Genes

GEPIA (Gene Expression Profiling Interactive Analysis), an online web server [20], was used to obtain the gene expression profiles of all the 5 hub genes in case of patients suffering from breast cancer. The survival analysis corresponding to these hub genes was obtained from SurvExpress [21]. The Kaplan–Meier (KM) plot used for visualizing the survival analyses of all the hub genes (prognostic biomarkers) is based on the univariate Cox regression analysis, which provides the risk score by categorizing the patients into low- and high-risk groups. 

## 3. Results

### 3.1. Determination of Differentially Expressed Genes through Statistical Analysis

**Figure 2:**
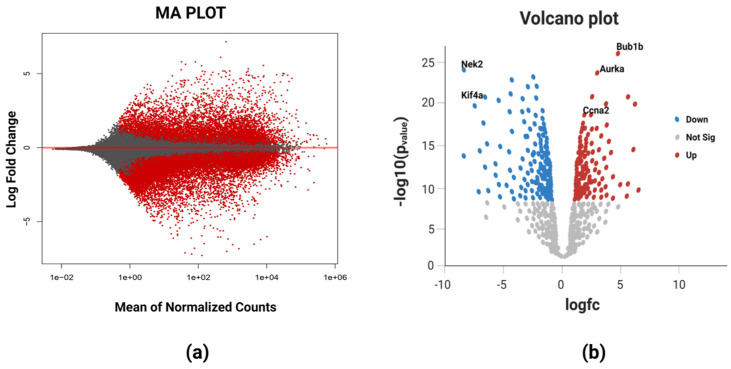
(**a**) MA plot of breast cancer representing the log fold change against mean expression using the DESeq2 dataset. The red dots corresponding to the positive *x*-axis represent upregulated differentially expressed genes, while those corresponding to the negative *x*-axis represent downregulated differentially expressed genes. (**b**) Volcano plot showing the most significant differentially expressed genes. The blue color dots on the left portion of the plot represent the downregulated DEGs, red color dots on the right portion of the plot represent upregulated DEGs, and white color dots at the bottom portion depict the non-significant DEGs. *BUB1B* is the most significant DEG based on its highest value of log fold change.

The RNA-Seq high-throughput analysis produced 2854 differentially expressed genes (DEGs) for breast cancer, out of which 1812 were upregulated and 1042 were downregulated. The upregulated and downregulated genes can be visualized using a Bland–Altman (MA) plot (Figure 2a). It could be evidenced from the figure that a greater number of DEGs was found in the positive *x*-axis showing more upregulated genes as compared to the downregulated genes in the negative *x*-axis. The volcano plot (Figure 2b) that provides the information about the most significant differentially expressed genes showed that all the five identified biomarkers in this study were upregulated as they all lie on the right portion of the plot shown by red dots. The blue dots represents the downregulated genes viz. *NEK2* (NIMA-related kinase 2) and *KIF4A* (Human kinase family member 4A), and these two lie on the left portion of the plot. The most significant differentially expressed gene among these five DEGs was *BUB1B* having the highest log fold change value in the deseq2 statistical analysis.

### 3.2. Investigation of the Protein–Protein Interaction Network (PPIN) Established the Hub Genes as Potential Prognostic Biomarkers

**Figure 3:**
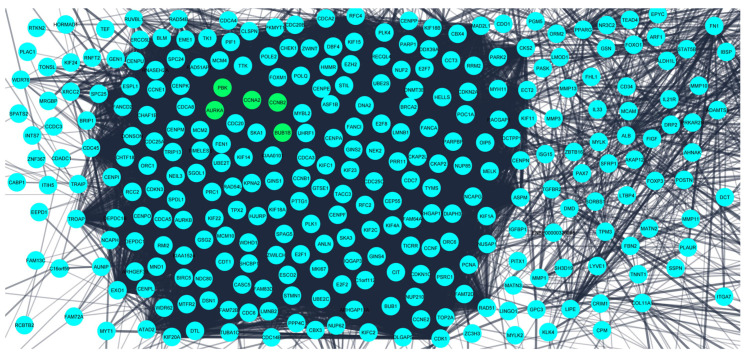
Protein–protein interaction network for the 824 differentially expressed genes obtained from STRING database. The confidence interval was >0.7. Nodes represent interacting proteins and the edges represent the interaction between these proteins. The 5 hub genes (potential prognostic biomarkers) are shown in green color.

**Figure 4:**
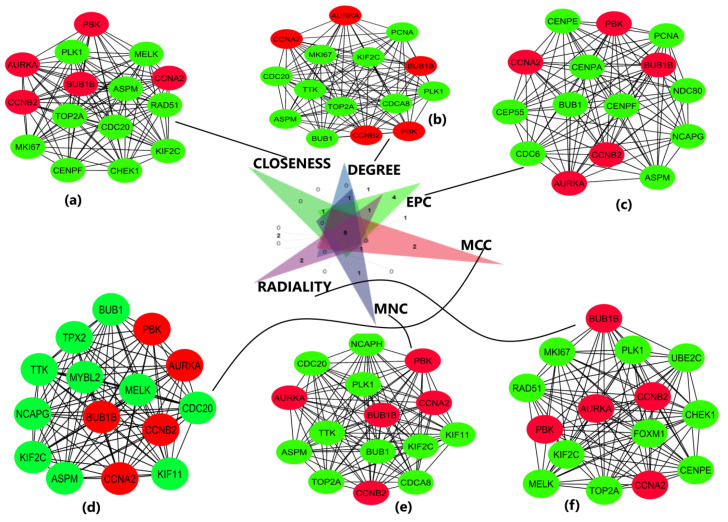
Important sub-networks and nodes obtained from cytohubba plug-in of Cytoscape software using six topological algorithms. The top 15 hub genes were evaluated in the PPI network using these six calculation methods. Red color circles represent the hub genes of interest in this study, and green color circles represent the adjoining genes obtained from network. (**a**) Sub-network obtained from closeness topological algorithm. The nodes in red represent the top-ranked hub genes. (**b**) Sub-network and hub genes obtained from degree topological algorithm. (**c**) Sub-network and hub genes obtained from EPC topological algorithm. (**d**) Sub-network and hub genes obtained from MCC topological algorithm. (**e**) Sub-network and hub genes obtained from MNC topological algorithm. (**f**) Sub-network and hub genes obtained from radiality topological algorithm. The hub genes are shown in red color in all 6 topologies.

The obtained DEGS were used for constructing the PPIN having 822 nodes and 12,145 edges. The average node degree was 29.5, the average local clustering coefficient was 0.453, and the PPI enrichment *p*-value was less than 1.0 × 10^−16^. The PPIN with the above characteristics is shown below (Figure 3). The five hub genes obtained from different topologies of cytohubba are *AURKA (Aurora Kinase A)*, *BUB1B (BUB1 Mitotic Checkpoint Serine/Threonine Kinase B), CCNA2 (Cyclin A2), CCNB2 (Cyclin B2), and PBK (PDZ Binding Kinase)* (Figure 4). The values and ranks of the hub genes in these algorithms are summarized in the table (Table 1). The five hub genes were upregulated in breast cancer, promoting tumorigenesis and metastasis.

### 3.3. Gene Oncology (GO) Component and KEGG Pathway Enrichment Analysis

**Figure 5:**
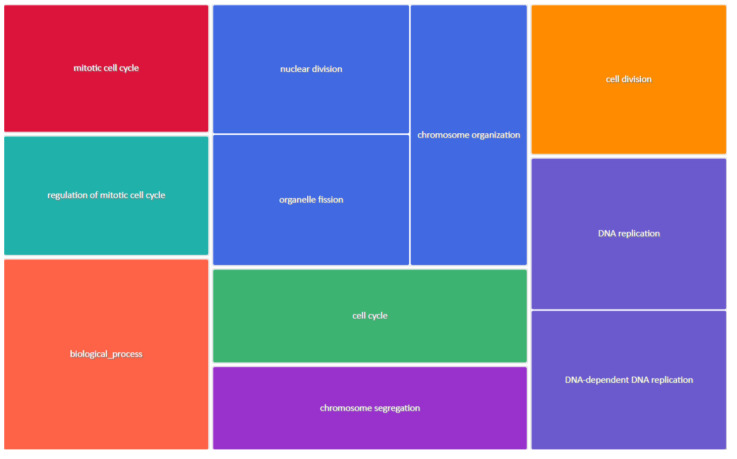
Tree map showing the Biological Processes (BP) based on *p*-values drawn from Revigo in which the hub genes were significantly enriched. The plot represents highly similar GO terms based on their respective *p*-values. Each rectangle in the tree map represents a single cluster representative. The different clusters are represented by different colors. The size of the rectangles is based on either the *p*-value or frequency of the GO terms in the GOA database.

The DAVID database provided the components and pathways in which the five hub genes participated and were enriched. The hub genes were found to be enriched in various biological processes such as the cell cycle, mitotic cell cycle, cell division, mitotic nuclear division, and chromosome segregation, and these are some of the most important processes that promotes tumorigenesis and the metastasis of breast cancer (Figure 5). The biological processes were ranked based on *p*-values, and these processes along-with their respective *p*-values are tabulated in the table (Table 2).

**Figure 6:**
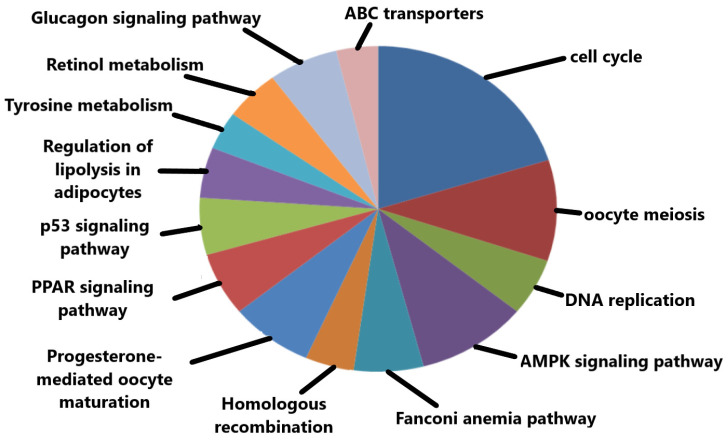
Important KEGG pathways in which the hub genes were significantly enriched.

The significant KEGG pathways based on *p*-values include oocyte meiosis, cell cycle, progesterone-mediated oocyte maturation, and p53 signaling pathway (Figure 6). Some of the top-ranked enriched KEGG pathways along with their respective *p*-values are tabulated below (Table 3).

### 3.4. Exploring the Epigenetic Regulation of Hub Genes through Promoter Methylation 

**Figure 7:**
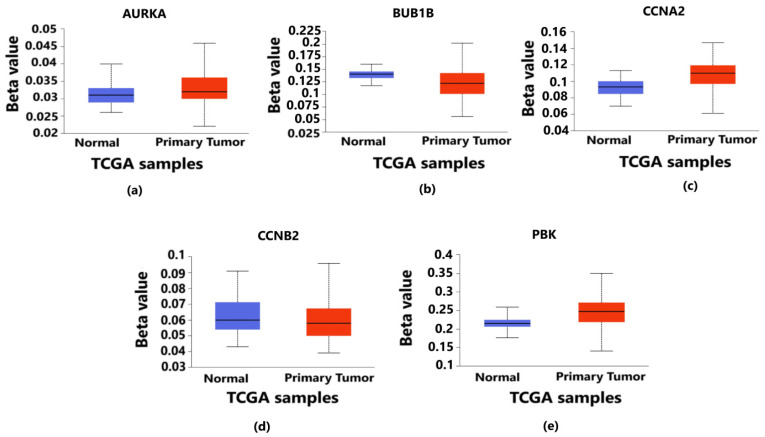
Level of promoter methylation corresponding to 5 hub genes in breast cancer. It provides the information about whether the overexpressed genes are hypermethylated or hypomethylated as both play vital roles in the progression and metastasis of breast cancer. The blue color box plot denotes normal TCGA samples without breast cancer, and the red color box plot represents the TCGA samples with breast cancer. (**a**) Methylation level of *AURKA* gene in which the methylation levels of tumor samples are higher than those of normal samples. (**b**) Methylation level of *BUB1B* gene in which the methylation levels of tumor samples are lower than those of normal samples. (**c**) Methylation level of *CCNA2* gene in which the methylation levels of tumor samples are higher than those of normal samples. (**d**) Methylation level of *CCNB2* gene in which the methylation levels of tumor samples are lower than those of normal samples. (**e**) Methylation level of *PBK* gene in which the methylation levels of tumor samples are higher than those of normal samples. In all the above cases, the *p*-value is less than 0.05 (*p* < 0.05).

Validation of promoter methylation through Student’s *t*-test between normal and primary tumor using the UALCAN database revealed that the promoter methylation level of *BUB1B* and *CCNB2* was lower than that of the normal samples in breast cancer, which indicates the higher expression of these hub genes (Figure 7b,d) (*p* < 0.05) in contrast to that of *AURKA, CCNA2* and *PBK* having a higher promoter methylation level than the normal samples (Figure 7a,c,e) (*p* < 0.05).

### 3.5. Findings of Genetic Alterations in Hub Genes

**Figure 8:**
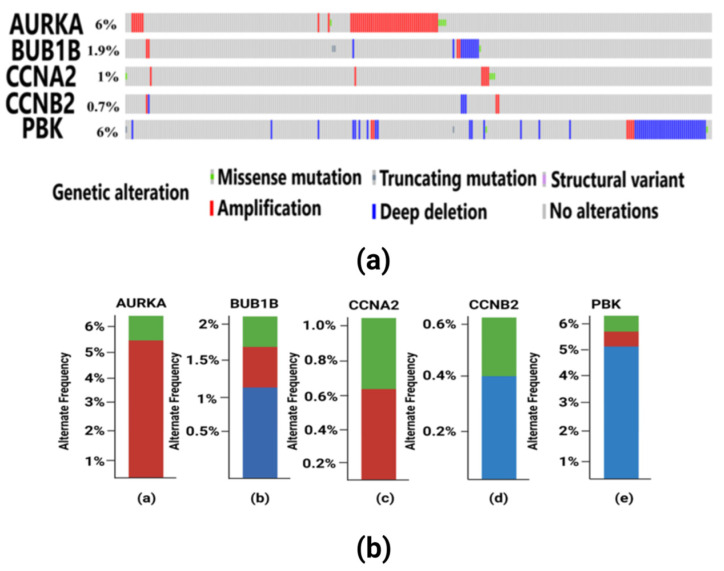
(**a**) Visualization of genetic alterations of hub genes in breast cancer using OncoPrint. Green color in the bar plot represents mutation, red color represents amplification, and blue color represents deep deletion in the cancer patient samples from TCGA. In this figure, *AURKA* have 6% genetic alteration having missense mutation in 5 TCGA patient samples and amplification in other samples. *BUB1B* have 1.9% genetic alterations which include missense and truncating mutations, amplification and deep deletion in some samples. *CCNA2* have 1% genetic alterations having missense and amplification. *CCNB2* consists of 0.7% alterations mainly consisting of amplification and deep deletion. PBK gene has 6% alterations having truncating and missense mutations, amplification and deep deletion in the patient samples. (**b**) Frequency of genetic alterations in hub genes in breast cancer. Red color indicates amplification, green color indicates mutations and blue color indicates deep deletion. (a) The *AURKA* gene has a more frequent occurrence of amplification in 5% of the samples and less frequent mutations in only 1% of the samples. (b) The *BUB1B* gene has more frequent deep deletion in 1% of the samples, which is followed by less frequent amplification and mutation each occurring in only 0.5% of the samples. (c) The *CCNA2* gene has a higher frequency of amplification in 0.6% of the samples and mutations in 0.4% of the samples. (d) The *CCNB2* gene has deep deletion having frequent occurrence in 0.4% of the samples and less frequency of occurrence of amplification in 0.2% of the patient samples. (e) The *PBK* gene has more genetic alterations due to deep deletion in 5% of the samples, which is followed by amplification in 0.8% of the samples and mutation in 0.2% of the samples, respectively.

**Figure 9:**
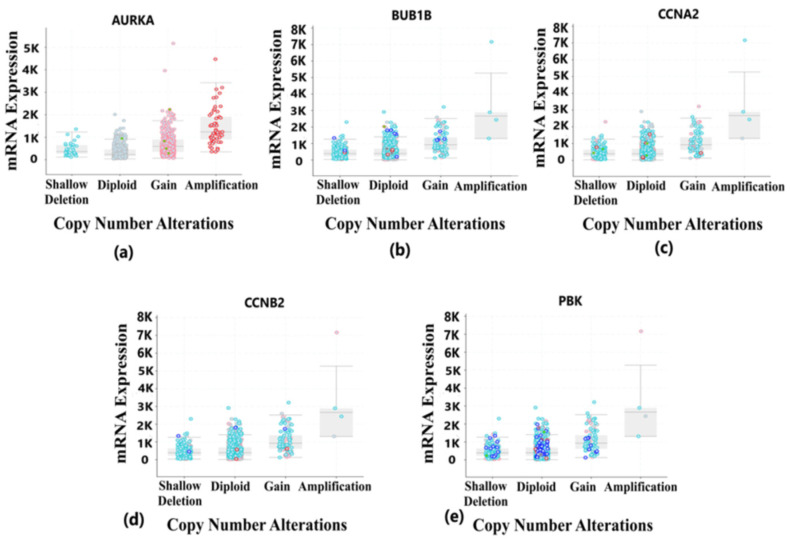
Copy number alteration deals with the deletions and amplification of genetic material fragments. This phenomenon is common in different cancer types and plays a vital role in the development and progression of cancer. This figure shows the copy number alterations in hub genes of BRCA. Light blue color dots represent shallow deletion, dark blue dots represent deep deletion, red dots represent gain, and green dots represent missense mutation. (**a**) Copy number alterations in the *AURKA* gene having most of the changes due to gain and amplification in the genetic materials. (**b**) Copy number alterations in *BUB1B* gene having most of the changes due to diploid, gain and shallow deletion in the genetic materials. (**c**) Copy number alterations in *CCNA2* gene having most of the changes due to shallow deletion, gain and diploid in the genetic materials. (**d**) Copy number alterations in *CCNB2* gene having most of the changes due to gain and amplification in the genetic materials. (**e**) Copy number alterations in *PBK* gene having most of the changes due to gain and amplification in the genetic materials.

Tumorigenesis mainly occurs due to irremediable mutations in cell structures. These mutations could be identified through genetic alteration analysis. The alterations may be in the form of missense mutation, splice mutation, deep deletion, truncating mutation, and amplification. In case of breast cancer, the percentage alteration of all the five hub genes varied from 0.7% to 6% (Figure 8a). The corresponding frequency of occurrence of the genetic alterations shows more frequency of amplification and mutations in all the five hub genes (Figure 8b). Copy number alterations for breast cancer show most of the alterations due to diploid, gain, and amplification. The *AURKA* gene was mostly affected due to amplification in the genetic materials, while the remaining four hub genes were mainly altered due to either gain, diploid or in some cases, deep deletion (Figure 9). The details of genetic alterations and copy number variations are summarized in the table below (Table 4). Almost all the mutations in these five hub genes were phosphorylated. 

### 3.6. Survival Analysis Validation of Prognostic Biomarkers

**Figure 10:**
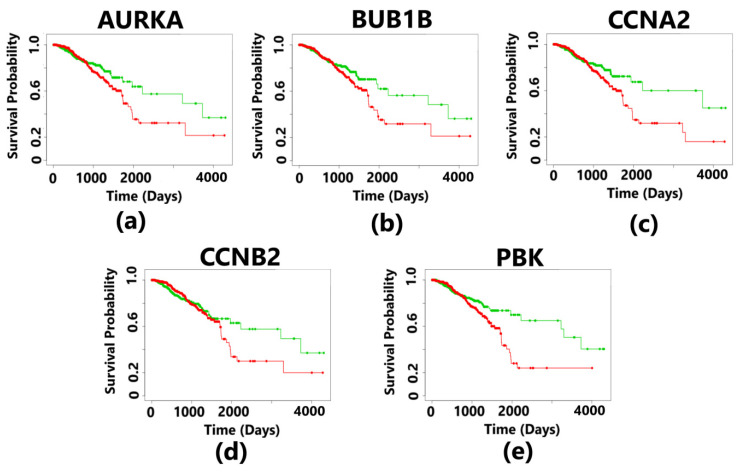
Kaplan–Meier plots showing the survival analysis corresponding to 5 hub genes in breast cancer. The patients were divided into high- and low-risk groups. The overexpression of all the hub genes resulted in the poor survival outcomes, which is less than 3 years for the patients suffering from breast cancer. The plot in green color indicates the survival of patients in the low-risk group, and the plot in red color represents the survival of patients in the high-risk group. The survival curves are statistically significant with a *p*-value less than 0.05 (*p*-value < 0.05). (**a**) The overexpression of the *AURKA* gene indicates a survival probability of 50% for patients in the low-risk group and 25% survival of the patients in the high-risk group. The positive control has 482 samples, and the negative control has 480 samples. (**b**) The overexpression of the *BUB1B* gene indicates a survival probability of 48% for patients in the low-risk group and 18% survival of the patients in the high-risk group. The positive control has 483 samples, and the negative control has 479 samples. (**c**) The overexpression of the *CCNA2* gene indicates a survival probability of 56% for patients in the low-risk group and 18% survival of the patients in the high-risk group. The positive control has 481 samples and the negative control also has 481 samples. (**d**) The overexpression of the *CCNB2* gene indicates a survival probability of 54% for patients in the low-risk group and 22% survival of the patients in the high-risk group. The positive control has 484 samples and the negative control has 478 samples. (**e**) The overexpression of the *PBK* gene indicates a survival probability of 52% for patients in the low-risk group and 23% survival of the patients in the high-risk group. The positive control has 482 samples and the negative control has 480 samples.

The aberrant expression of *AURKA, BUB1B, CCNA2, CCNB2*, and *PBK* resulted in a poorer survival rate of breast cancer patients in the high-risk group having a survival rate of less than 2 years. The survival curves are statistically significant with a *p*-value < 0.05, and this *p*-value is based on a log-rank test (Wilcoxon test). The median survival rate was less than 2 years for all the five hub genes (Figure 10). For each patient, the risk score was calculated and ranking was completed accordingly in the TCGA datasets. Patients were then divided into a high-risk group and a low-risk group. The hazard ratio of the hub genes indicates the risk associated with the survival of the patients (Table 5). The survival rate of the patients was found to be the least in case of overexpressed *BUB1B* having a survival probability of low-risk patients of only 48%, while those in the high-risk group had a survival probability only 18%, and the hazard ratio was also the highest as compared to that of other hub genes.

## 4. Discussion

Cancer is a dreadful disease, and it costs millions of lives every year, more specifically, breast cancer, which is common among women across the globe. Proper awareness of the biological insight and better understanding of this cancer type through complex networks and signaling might help in the early diagnosis and treatment of breast cancer [22]. This in-depth understanding was studied in this research work through transcriptome analysis. The transcriptome analysis paved the way to identify the overexpressed differentially expressed genes that could be potential prognostic biomarkers of breast cancer that could help in prohibiting the tumorigenesis and metastasis of breast cancer. The identification of patients with high risk of breast cancer is important to provide effective and specific treatment. These above-discussed gene expression profiling concepts will aid in the identification of novel prognostic biomarkers with greater accuracy [23]. The identified biomarkers could regulate the analysis of survival of the patients using a Kaplan–Meier plot based on which the survival probability could be predicted, thereby proving these biomarkers as potential therapeutics involved in the identification of differentially expressed genes through the transcriptomic approach. Subsequently, we obtained the protein–protein interaction network by utilizing these differentially expressed genes to identify the most prominent hub genes (prognostic biomarkers) viz. *AURKA, BUB1B, CCNA2, CCNB2,* and *PBK*, and these hub genes obtained were found to be upregulated (based on log_2_fold change value) in breast cancer. Pathway enrichment analysis further showed the biological processes and pathways in which these biomarkers were enriched. The survival analysis predicted poorer prognosis of the patients suffering from these cancer types due to the overexpression of these prognostic biomarkers. The promoter methylation validation showed these biomarkers to be hypomethylated in breast cancer and could be a probable cause of spread of breast cancer and development [24]. Moreover, the analysis of genetic alterations that provides information pertaining to variations in prognostic biomarkers could furnish how these changes aid in the progression and metastasis of cancer and its detection, diagnosis and prognosis [25]. This genetic alterations in the form of mutations and copy number alterations provided an in-depth understanding of genetic changes in the biomarkers that resulted in the tumorigenesis and metastasis of breast cancer in patients.

The five potential prognostic biomarkers, i.e., *Aurora Kinase A (AURKA), BUB1 Mitotic checkpoint serine/threonine kinase B (BUB1B), Cyclin A2 (CCNA2), Cyclin B2 (CCNB2), and PDZ binding kinase (PBK)* were upregulated in breast cancer. These genes were enriched in some of the important biological processes that include mitotic cell cycle, cell division, regulation of mitotic cell cycle, and chromosome segregation. Chromosome segregation is a particularly important biological process due to its relation in the development and progression of cancer. The errors introduced in chromosome segregation during mitosis lead to chromosomal instability, which is responsible for tumorigenesis, cancer metastasis and poor prognosis in cancer patients [26]. The abnormal count of chromosomes due to genomic instabilities plays a pivotal role in tumorigenesis and cancer metastasis [27]. The important KEGG pathways that participated in tumorigenesis and metastasis showed the enrichment of the biomarkers in the p53 signaling pathway, cell cycle, oocyte meiosis, progesterone-mediated oocyte maturation, glucagon signaling pathway, and PPAR signaling pathway. In this study, it was observed that the potential biomarkers are overrepresented in the cell cycle KEGG pathway. This improper regulation of cell cycle may result in uncontrolled cell multiplication, and this phenomenon leads to tumorigenesis and cancer metastasis [28]. The two other important KEGG pathways in which the biomarkers were enriched are oocyte meiosis and progesterone-mediated oocyte maturation. In the meiosis process, two more rounds of chromosome segregation (Meiosis I and Meiosis II) are followed by a single round of DNA replication [29]. At G2 of meiosis I, oocytes are naturally arrested, and this arrest is broken by the encounter to the progesterone, which is a steroid hormone. This persuades the maturation of the oocyte and the two meiotic division cycles process to be resumed [29]. So, it may be inferred that the cell cycle process might be affected due to an abnormal regulation of meiosis and oocyte maturation. Moreover, this change in cell cycle had a negative impact on normal activities in the human body resulting in increased risks of suffering from different types of cancer. Moreover, two genes viz. *NEK2* and *KIF4A* were downregulated. Although *NEK2* has been found to be downregulated in this study, it has been reported as overexpressed in one of the studies [30,31]. Likewise, the *KIF4A* gene is also downregulated in the following study and found to have a strong correlation with malignant breast cancer. Hence, it could be a prognostic biomarker for this cancer type [32].

*AURKA*, an oncogene from the serine/threonine kinase family, is responsible for activating the process of cell division through mitosis regulation and promoting tumorigenesis and metastasis in different cancer types, and this property qualifies *AURKA* as a potential target in cancer treatment [33,34]. This gene is related to cell cycle progression, and hence, its inhibition might lead to the regression of breast cancer [35]. This gene was hypomethylated (beta value 0.034), which causes genetic instability and is the primary reason for the development and metastasis of breast cancer. The mutation is mainly missense type and occurred at five different mutation sites (S98N, S4Y, S89C, A81V, and L26V), and also this gene has been identified in amplified regions due to gene amplification, resulting in genetic alterations and phosphorylation. This post-transcriptional modification affects many significant pathways in which the *AURKA* gene was enriched, such as the cell cycle, and played a key role in breast cancer growth and metastasis. So, this altered phosphorylation could be a potential target for the development of suitable anti-cancer drugs that can inhibit the progression and metastasis of breast cancer [36]. The diploid and gain copy number alterations found in this gene also played a role in the development and progression of breast cancer [37]. The statistically significant (*p*-value < 0.05) survival analysis showed poor prognosis in case of *AURKA* having a hazard ratio > 1 (1.32). The patients in the low-risk group have a higher survival probability (50%) than those in the high-risk groups with a survival probability of 25%. The overexpression and poor survival rate indicate this gene to be a potential predictive biomarker for the early detection and diagnosis of metastatic breast cancer.

The *BUB1B* gene plays a vital role in encoding a kinase which is involved in the spindle checkpoint function, resulting in many cancer forms. In breast cancer metastasis, the chromosomal instability was found to be the main cause, and this defect pertains to imperfection in mitotic spindle checkpoints. This process is related to the overexpression of the *BUB1B* gene [38]. The *BUB1B* gene also caused a decrease in the survival probability of the patients suffering from breast cancer and resulting in metastasis in another study [39].

The DNA methylation showed a higher expression of this gene in breast cancer (beta value: 0.125). The missense mutation at was formed at two sites (Q460E and L669P) and a nonsense mutation was formed at another single site (S564*). Another genetic alteration was amplification with a frequency 0.32% in the breast cancer patients affected due to the overexpression of this gene in contrast to mutation (0.39%) and deep deletion (1.12%). The copy number alterations were gain and diploid can, and these are most prominent in producing cancer. The survival analysis of *BUB1B* showed a survival probability of 48% in case of low-risk group patients and 18% in case of high-risk group patients. The hazard ratio was 1.85, which was very high and proved the overexpression and poor prognosis of this gene to be a potential prognostic biomarker for breast cancer.

*CCNA2* is a protein-coding gene which plays a prominent role in the progression and distant metastasis of breast cancer and could be a biomarker [40]

*CCNA2,* which was overexpressed in case of breast cancer and has an oncogenic role in cancer [41], participates in the tumorigenesis and metastasis of breast cancer. The promoter methylation showed that the *CCNA2* gene was hypomethylated, leading to the speedy tumor progression and metastasis. The genetic alterations that are involved in the overexpression of *CCNA2* include mutation and amplification. There are missense mutation at four mutation sites (R112C, L315P, M189I, and V85F). The other genetic alteration, i.e., amplification was related to an increased growth of breast cancer cells and further assisted in its metastasis due to the upregulation of the *CCNA2* gene. The copy number alterations that were associated with this gene include diploid and shallow deletion can, and both of these are already discussed to promote tumor growth and metastasis. The survival analysis demonstrated that the overall survival probability of the patients in the low-risk group was 56% compared to the high-risk group, where it was only 18%. The hazard ratio was < 1 (0.49), and this showed that the overexpression of this gene was comparatively less effective in case of breast cancer as compared to other biomarkers. However, the survival probability, particularly in the case of the high-risk group, was associated with poor prognosis, and hence, this gene could be a significant predictive biomarker for the diagnosis and inhibition of breast cancer tumorigenesis and metastasis.

The overexpression and oncogenic role of the *CCNB2* gene was responsible for the metastasis of breast cancer. This overexpression of this gene had an adverse effect on the normal functioning of the cells, and hence, the breast cancer cells metastasized. Moreover, the promoter methylation level showed a higher expression level of this gene in case of metastatic breast cancer (beta value–0.06). The genetic alterations consisted of amplification and deep deletion and took part in the promotion of tumor growth and metastasis. The survival analysis shows that the expression level of this gene was in a controlled manner. The survival probability was 54% in the low-risk group of patients, and those in the high-risk group had a survival probability of 22%. The genetic alteration analysis showed that only gene amplification participated in producing the genomic instability of this gene. This higher expression of *CCNB2* as shown by the results of promoter methylation and poor prognosis obtained from the survival analysis demonstrated the efficacy of this gene to be a suitable candidate for the prediction, diagnosis and treatment of HCC.

The *PBK* gene, which was also overexpressed, was found to have an association with the poor survival of patients in different cancer, and this made *PBK* a suitable prognostic biomarker and a potential therapeutic target [41].

In the case of breast cancer, the *PBK* gene was found to be overexpressed, and this resulted in the progression and probable metastasis of breast cancer to form GBM and HCC. In one of the latest studies, it was reported that the overrepresentation of the *PBK* gene resulted in a poor prognosis of patients suffering from breast cancer [42]. The promoter methylation level validated the lower expression of this gene in case of breast cancer (beta value: 0.25). The genetic alteration study further demonstrated the involvement of amplification, mutation, and deep deletion in producing the overexpression of the *PBK* gene. The missense mutation at two mutation sites (E203K and F40L) and nonsense mutation at a single mutation site (E295*) showed the genomic instability that caused the growth and metastasis of breast cancer. In addition to mutation, the other two alterations that were responsible for overexpression include amplification and deep deletion (FS deletion at K18Efs*50). The phosphorylation post-translational modification was also altered, resulting in further progression of cancer. The survival analysis showed that the hazard ratio was 1.26. The survival probability of patients in the low-risk group was 52%, while that in the high-risk group was 23%. The poor prognosis of this gene qualified it to be a suitable indicator for the prediction and diagnosis of breast cancer metastasis. Although our results suggest that copy number alterations are associated with the changes in gene expression in the five hub genes identified in this study, there are some genes such as CDK4 and MYC, which can be amplified without resulting in increased mRNA levels [43,44]. Further research is needed to investigate the complex relationship between copy number alterations and gene expression. Understanding the mechanisms that regulate gene expression in the context of copy number alterations may help in identify additional hub genes and developing more effective therapeutic strategies for cancer treatment.

Therefore, current standard-of-care biomarkers such as estrogen receptor (ER), progesterone receptor (PR), and human epidermal growth factor receptor 2 (HER2) provide important prognostic and predictive information in case of breast cancer [45]. For example, HER2-positive breast cancer is typically treated with targeted therapy, such as trastuzumab, while ER-positive breast cancer may be treated with endocrine therapy [46]. In addition, Oncotype DX is a widely used biomarker that provides prognostic information for patients with early-stage breast cancer and can help in guide treatment decisions [47]. However, these biomarkers have limitations. For example, not all breast cancers express HER2 or have hormone receptor expression, and some patients may have tumors that are HER2-negative and ER-negative, making them ineligible for targeted therapy or endocrine therapy [48]. Moreover, although Oncotype DX provides important prognostic information, it is expensive and not universally available. It may not provide information beyond the basic clinical and pathological factors already guiding treatment decisions.

Our identified biomarkers, AURKA, BUB1B, CCNA2, CCNB2, and PBK, can potentially provide additional prognostic and predictive information beyond current standard-of-care biomarkers, including Oncotype DX.

Furthermore, they were identified through an integrated approach of transcriptome and pathway enrichment analysis, providing a more comprehensive understanding of the underlying biology of breast cancer. However, further validation of these biomarkers in future studies is needed to determine their clinical utility in guiding treatment decisions and improving patient outcomes [49].

## 5. Conclusions

The present study provided five potential prognostic biomarkers viz. *AURKA*, *BUB1B*, *CCNA2*, *CCNB2*, and *PBK* through the integrated approach of transcriptome and pathway enrichment analysis. This will aid in the early diagnosis and treatment of breast cancer and could probably improve the survival analysis of the patients. The proper designing of potential inhibitors for these biomarkers will help immensely in suppressing the tumorigenesis and metastasis of breast cancer.

## Figures and Tables

**Table 1:** The values of hub genes for various topological algorithms of cytohubba.

Hub Genes	Closeness	Degree	EPC	MCC	MNC	Radiality
*AURKA*	192.6	106	38.26	1.65 × 10⁵⁷	106	6.67
*BUB1B*	194.7	112	43.76	1.65 × 10⁵⁷	112	6.62
*CCNA2*	200.4	109	38.26	1.65 × 10⁵⁷	109	6.73
*CCNB2*	190.6	110	41.25	1.65 × 10⁵⁷	110	6.52
*PBK*	199.5	115	42.23	1.65 × 10⁵⁷	115	6.61

**Table 2:** Top 10 significantly enriched biological processes along with their respective *p*-values.

Biological Process	*p*-Value
GO:0000278~Mitotic Cell Cycle	1.13 × 10^−43^
GO:1903047~Mitotic Cell Cycle Process	2.43 × 10^−43^
GO:0022402~Cell Cycle Process	7.17 × 10^−40^
GO:0007049~Cell Cycle	1.97 × 10^−35^
GO:0000280~Nuclear Division	1.99 × 10^−33^
GO:0051301~Cell Division	3.12 × 10^−33^
GO:0007059~Chromosome Segregation	4.28 × 10^−33^
GO:0000819~Sister Chromatid Segregation	5.08 × 10^−33^
GO:0007067~Mitotic Nuclear Division	3.20 × 10^−32^
GO:0048285~Organelle Fission	1.05 × 10^−31^

**Table 3:** Top 10 significantly enriched KEGG pathways with their respective *p*-values.

KEGG Pathways	*p*-Value
hsa04110:Cell Cycle	1.71 × 10⁻¹⁵
hsa04114:Oocyte Meiosis	1.27 × 10⁻⁴
hsa03030:DNA Replication	1.59 × 10⁻⁴
hsa04152:AMPK Signaling Pathway	4.01 × 10⁻⁴⁰
hsa03460:Fanconi Anemia Pathway	5.36 × 10⁻⁴
hsa03440:Homologous Recombination	0.001599
hsa04914:Progesterone-Mediated Oocyte Maturation	0.001748
hsa03320:PPAR Signaling Pathway	0.010183
hsa04115:p53 Signaling Pathway	0.012470
hsa04923:Reguation of Lipolysis in Adipocytes	0.011456

**Table 4:** Table summarizing the information related to genetic alterations in breast cancer.

S. No.	Gene Name	Types of Genetic Alterations (%)	Post Translational Modifications (PTMs)	Mutation Type	Mutation Site	Copy Number Alteration
1	*AURKA*	Mutation (0.24%) Amplification (5.29%)	Phosphorylation	Missense	S98N	Diploid
			Phosphorylation	Missense	S4Y	Diploid
			Phosphorylation	Missense	S89C	Gain
			NA	Missense	A81V	Gain
			NA	Missense	L26V	Gain
2	*BUB1B*	Mutation (0.39%) Amplification (0.32%) Deep Deletion (1.12%)	NA	Missense	Q460E	Gain
			NA	Missense	L669P	Gain
			NA	Nonsense	S564*	Diploid
			NA	FS del	D989Mfs*13	Diploid
3	*CCNA2*	Mutation (0.49%) Amplification (0.62%)	NA	Missense	R112C	Diploid
			NA	Missense	L315P	Shallow Deletion
			NA	Missense	M189I	Shallow Deletion
			NA	Missense	V85F	Diploid
4	*CCNB2*	Amplification (0.29%) Deep Deletion (0.41%)	NA	NA	NA	NA
5	*PBK*	Mutation (0.40%) Deep Deletion (5.1%) Amplification (0.8%)	Phosphorylation	Missense	E203K	Shallow Deletion
				Missense	F40L	Shallow Deletion
				Nonsense	E295*	Diploid
				FS del	K18Efs*50	Gain

**Table 5:** Table showing the survival analysis results of hub genes in breast cancer.

S. No.	Gene	Hazard Ratio (CI)
1	*AURKA*	1.32 (0.91–2.42)
2	*BUB1B*	1.85 (1.32–2.92)
3	*CCNA2*	0.49 (0.40–0.95)
4	*CCNB2*	0.62 (0.41–1.01)
5	*PBK*	1.26 (0.90–2.13)

## Data Availability

The raw data for the solid normal samples and primary tumor were obtained from The Cancer Genome Atlas (TCGA) (The Cancer Genome Atlas Program (TCGA)—NCI).

## References

[1] Zeng X., Shi G., He Q., Zhu P. (2021). Screening and Predicted Value of Potential Biomarkers for Breast Cancer Using Bioinformatics Analysis. Sci. Rep..

[2] World Health Organization (2020). Breast Cancer.

[3] Pan Y., Liu G., Yuan Y., Zhao J., Yang Y., Li Y. (2017). Analysis of Differential Gene Expression Profile Identifies Novel Biomarkers for Breast Cancer. Oncotarget.

[4] Deng J.-L., Xu Y.-H., Wang G. (2019). Identification of Potential Crucial Genes and Key Pathways in Breast Cancer Using Bioinformatic Analysis. Front. Genet..

[5] Kim G.-E., Kim N.I., Lee J.S., Park M.H., Kang K. (2020). Differentially Expressed Genes in Matched Normal, Cancer, and Lymph Node Metastases Predict Clinical Outcomes in Patients with Breast Cancer. Appl. Immunohistochem. Mol. Morphol..

[6] Ellsworth R.E., Field L.A., Love B., Kane J.L., Hooke J.A., Shriver C.D. (2011). Differential Gene Expression in Primary Breast Tumors Associated with Lymph Node Metastasis. Int. J. Breast Cancer.

[7] Zhao X., Yan H., Yan X., Chen Z., Zhuo R. (2022). A Novel Prognostic Four-Gene Signature of Breast Cancer Identified by Integrated Bioinformatics Analysis. Dis. Markers..

[8] Jin H., Huang X., Shao K., Li G., Wang J., Yang H., Hou Y. (2019). Integrated Bioinformatics Analysis to Identify 15 Hub Genes in Breast Cancer. Oncol. Lett..

[9] Ren Y., Deng R., Zhang Q., Li J., Han B., Ye P. (2020). Bioinformatics Analysis of Key Genes in Triple Negative Breast Cancer and Validation of Oncogene PLK1. Ann. Transl. Med..

[10] Dobin A., Davis C.A., Schlesinger F., Drenkow J., Zaleski C., Jha S., Batut P., Chaisson M., Gingeras T.R. (2013). STAR: Ultrafast Universal RNA-seq Aligner. Bioinformatics.

[11] Liao Y., Smyth G.K., Shi W. (2014). FeatureCounts: An Efficient General Purpose Program for Assigning Sequence Reads to Genomic Features. Bioinformatics.

[12] Love M.I., Huber W., Anders S. (2014). Moderated Estimation of Fold Change and Dispersion for RNA-seq Data with DESeq2. Genome Biol..

[13] Szklarczyk D., Gable A.L., Nastou K.C., Lyon D., Kirsch R., Pyysalo S., Doncheva N.T., Legeay M., Fang T., Bork P. (2021). The STRING Database in 2021: Customizable Protein-Protein Networks, and Functional Characterization of User-Uploaded Gene/Measurement Sets. Nucleic Acids Res..

[14] Shannon P., Markiel A., Ozier O., Baliga N.S., Wang J.T., Ramage D., Amin N., Schwikowski B., Ideker T. (2003). Cytoscape: A Software Environment for Integrated Models of Biomolecular Interaction Networks. Genome Res..

[15] Bardou P., Mariette J., Escudié F., Djemiel C., Klopp C. (2014). Jvenn: An Interactive Venn Diagram Viewer. BMC Bioinform..

[16] Dennis G., Sherman B.T., Hosack D.A., Yang J., Gao W., Lane H.C., Lempicki R.A. (2003). DAVID: Database for Annotation, Visualization, and Integrated Discovery. Genome Biol..

[17] Supek F., Bošnjak M., Škunca N., Smuc T. (2011). REVIGO Summarizes and Visualizes Long Lists of Gene Ontology Terms. PLoS ONE.

[18] Chandrashekar D.S., Bashel B., Balasubramanya S.A.H., Creighton C.J., Ponce-Rodriguez I., Chakravarthi B.V., Varambally S. (2017). UALCAN: A Portal for Facilitating Tumor Subgroup Gene Expression and Survival Analyses. Neoplasia.

[19] Gao J., Aksoy B.A., Dogrusoz U., Dresdner G., Gross B., Sumer S.O., Sun Y., Jacobsen A., Sinha R., Larsson E. (2013). Integrative analysis of complex cancer genomics and clinical profiles using the cBioPortal. Sci. Signal..

[20] Tang Z., Li C., Kang B., Gao G., Li C., Zhang Z. (2017). GEPIA: A web server for cancer and normal gene expression profiling and interactive analyses. Nucleic Acids Res..

[21] Aguirre-Gamboa R., Gomez-Rueda H., Martínez-Ledesma E., Martínez-Torteya A., Chacolla-Huaringa R., Rodriguez-Barrientos A., Tamez-Pena J.G., Treviño V. (2013). SurvExpress: An Online Biomarker Validation Tool and Database for Cancer Gene Expression Data Using Survival Analysis. PLoS ONE.

[22] Vishnubalaji R., Nair V.S., Ouararhni K., Elkord E., Alajez N.M. (2019). Integrated Transcriptome and Pathway Analyses Revealed Multiple Activated Pathways in Breast Cancer. Front. Oncol..

[23] Lee K.K., Chng W.J., Jha S. (2018). Prognostic Biomarkers for Breast Cancer Metastasis. Cancer Metastasis.

[24] Hoffmann M.J., Schulz W. (2005). Causes and Consequences of DNA Hypomethylation in Human Cancer. Biochem. Cell Biol..

[25] Herceg Z., Hainaut P. (2007). Genetic and Epigenetic Alterations as Biomarkers for Cancer Detection, Diagnosis and Prognosis. Mol. Oncol..

[26] Bakhoum S.F., Cantley L.C. (2018). The Multifaceted Role of Chromosomal Instability in Cancer and Its Microenvironment. Cell.

[27] Thompson S.L., Compton D.A. (2011). Chromosomes and Cancer Cells. Chromosom. Res..

[28] Novitasari D., Jenie R.I., Kato J.-Y., Meiyanto E. (2021). The Integrative Bioinformatic Analysis Deciphers the Predicted Molecular Target Gene and Pathway from Curcumin Derivative CCA-1.1 against Triple-Negative Breast Cancer (TNBC). J. Egypt. Natl. Cancer Inst..

[29] Mehlmann L.M. (2012). Signaling for Meiotic Resumption in Granulosa Cells, Cumulus Cells, and Oocyte. Oogenesis.

[30] Mahrous E., Yang Q., Clarke H.J. (2012). Regulation of Mitochondrial DNA Accumulation during Oocyte Growth and Meiotic Maturation in the Mouse. Reproduction.

[31] Shao H., Li R., Ma C., Chen E., Liu X.J. (2013). Xenopus Oocyte Meiosis Lacks Spindle Assembly Checkpoint Control. J. Cell Biol..

[32] Kokuryo T., Yokoyama Y., Yamaguchi J., Tsunoda N., Ebata T., Nagino M. (2019). NEK2 Is an Effective Target for Cancer Therapy with Potential to Induce Regression of Multiple Human Malignancies. Anticancer Res..

[33] Xue D., Cheng P., Han M., Liu X., Xue L., Ye C., Wang K., Huang J. (2018). An Integrated Bioinformatical Analysis to Evaluate the Role of KIF4A as a Prognostic Biomarker for Breast Cancer. OncoTargets Ther..

[34] Du R., Huang C., Liu K., Li X., Dong Z. (2021). Targeting AURKA in Cancer: Molecular Mechanisms and Opportunities for Cancer Therapy. Mol. Cancer..

[35] Siggelkow W., Boehm D., Gebhard S., Battista M., Sicking I., Lebrecht A., Solbach C., Hellwig B., Rahnenführer J., Koelbl H. (2012). Expression of Aurora Kinase A is Associated with Metastasis-Free Survival in Node-Negative Breast Cancer Patients. BMC Cancer.

[36] Wheater M.J., Johnson P.W., Blaydes J.P. (2010). The Role of MNK Proteins and eIF4E Phosphorylation in Breast Cancer Cell Proliferation and Survival. Cancer Biol. Ther..

[37] Baslan T., Kendall J., Volyanskyy K., McNamara K., Cox H., D’Italia S., Ambrosio F., Riggs M., Rodgers L., Leotta A. (2020). Novel Insights into Breast Cancer Copy Number Genetic Heterogeneity Revealed by Single-Cell Genome Sequencing. Elife.

[38] Yuan B., Xu Y., Woo J.-H., Wang Y., Bae Y.K., Yoon D.-S., Wersto R.P., Tully E., Wilsbach K., Gabrielson E. (2006). Increased Expression of Mitotic Checkpoint Genes in Breast Cancer Cells with Chromosomal Instability. Clin. Cancer Res..

[39] Koyuncu D., Sharma U., Goka E.T., Lippman M.E. (2021). Spindle Assembly Checkpoint Gene BUB1B Is Essential in Breast Cancer Cell Survival. Breast Cancer Res. Treat..

[40] Gan Y., Li Y., Li T., Shu G., Yin G. (2018). CCNA2 Acts as a Novel Biomarker in Regulating the Growth and Apoptosis of Colorectal Cancer. Cancer Manag. Res..

[41] Xu M., Xu S. (2019). PBK/TOPK Overexpression and Survival in Solid Tumors: A PRISMA-Compliant Meta-Analysis. Medicine.

[42] Qiao L., Ba J., Xie J., Zhu R., Wan Y., Zhang M., Jin Z., Guo Z., Yu J., Chen S. (2022). Overexpression of PBK/TOPK Relates to Poor Prognosis of Patients with Breast Cancer: A Retrospective Analysis. World J. Surg. Oncol..

[43] Sauter E.R., Yeo U.C., von Stemm A., Zhu W., Litwin S., Tichansky D.S., Pistritto G., Nesbit M., Pinkel D., Herlyn M. (2002). Cyclin D1 Is a Candidate Oncogene in Cutaneous Melanoma. Cancer Res..

[44] Pollock P., Harper U.L., Hansen K.S., Yudt L.M., Stark M., Robbins C.M., Moses T.Y., Hostetter G., Wagner U., Kakareka J. (2003). High Frequency of BRAF Mutations in Nevi. Nat. Genet..

[45] Tarighati E., Keivan H., Mahani H. (2023). A Review of Prognostic and Predictive Biomarkers in Breast Cancer. Clin. Exp. Med..

[46] Swain S.M., Shastry M., Hamilton E. (2023). Targeting HER2-positive breast cancer: Advances and future directions. Nat. Rev. Drug Discov..

[47] Moore J., Wang F., Pal T., Reid S., Cai H., Bailey C.E., Zheng W., Lipworth L., Shu X.O. (2022). Oncotype DX risk recurrence score and total mortality for early-stage breast cancer by race/ethnicity. Cancer Epidemiol. Biomark. Prev..

[48] Furrer D., Sanschagrin F., Jacob S., Diorio C. (2015). Advantages and disadvantages of technologies for HER2 testing in breast cancer specimens. Am. J. Clin. Pathol..

[49] Sagini K., Urbanelli L., Buratta S., Leonardi L., Emiliani C. (2017). Nanovesicles from plants as edible carriers of bioactive compounds. AgroLife Sci. J..

